# The prevalence and prognostic value of *KRAS* co‐mutation subtypes in Chinese advanced non‐small cell lung cancer patients

**DOI:** 10.1002/cam4.2682

**Published:** 2019-11-10

**Authors:** Dongjing Cai, Chengping Hu, Li Li, Shichao Deng, Jing Yang, Han Han‐Zhang, Min Li

**Affiliations:** ^1^ Department of Respiratory Medicine Xiangya Hospital Central South University Changsha Hunan China; ^2^ National Clinical Research Center for Geriatric Disorders Xiangya Hospital Central South University Changsha Hunan China; ^3^ Burning Rock Biotech Guangzhou International Biotech Island Guangzhou China; ^4^ Center for Molecular Medicine Xiangya Hospital Central South University Changsha Hunan China; ^5^ Key Laboratory of Molecular Radiation Oncology of Hunan Province Changsha Hunan China

**Keywords:** co‐mutation, Chinese, heterogeneity, *KRAS*, non‐small cell lung cancer

## Abstract

**Objective:**

*KRAS* mutation plays a critical role in the initiation and development of non‐small cell lung cancer (NSCLC). KRAS‐mutant patients exhibit diverse response to chemotherapy. *KRAS* co‐mutation subtypes and their prognosis value in advanced Chinese NSCLC patients remain largely elusive.

**Methods:**

A total of 1126 Chinese advanced NSCLC patients from Xiangya hospital were screened by capture‐based ultra‐deep sequencing for KRAS mutation between January 2015 and December 2016. Survival analyses were performed using Kaplan‐Meier analysis.

**Results:**

Among the patients screened, 84 cases were detected with *KRAS* mutation (7.5%). All of them were non‐squamous NSCLC and received pemetrexed plus platinum as the first‐line treatment. The most frequent *KRAS* co‐mutation genes were *TP53* (29%), *TP53*/*LKB1* (19%), and *LKB1* (14%). Our data revealed that patients with *KRAS* co‐mutation had poorer prognosis in comparison with those harboring single *KRAS* mutation. Furthermore, patients with KPL (*KRAS* mutated with *TP53* and *LKB1*) subtype, which was a novel subtype, had the shortest progression‐free survival (PFS) in all types of *KRAS* co‐mutation patients (*P* < .0001). The PFS and overall survival (OS) of patients with *KRAS^G12D^* mutation were inferior than those with *KRAS^G12C^* mutation or *KRAS^G12V^*mutation. Patients in *KRAS^G>T^* type had significantly longer survival than those in *KRAS^G>C^* type or *KRAS^G>A^* type.

**Conclusion:**

Our study revealed that concurrent genomic alterations can further stratify KRAS‐mutant lung adenocarcinoma patients into various subgroups with distinctive therapeutic responses and differential survival outcomes. The KPL is a novel and less responsive subtype among *KRAS*‐mutated NSCLC, and further investigation of effective treatment for this subtype is warranted.

## Graphical abstract

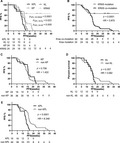

## 1 INTRODUCTION

Lung cancer causes 1.6 million death each year globally, while non‐small cell lung cancer (NSCLC) composes of 85% of all lung cancer. Therefore, tremendous efforts have been invested in elucidating the molecular mechanisms of NSCLC development and therapeutic targets.^1^, ^2^, ^3^ With the advancements in molecular biology and next‐generation sequencing technologies, numerous therapeutic targets were discovered, which subsequently revolutionized the management of NSCLC.^4^, ^5^ Approximately, 10% of NSCLC patients harbor *KRAS* mutation, which lacks effective therapeutic agents.^6^, ^7^, ^8^ One of the reason is that *KRAS* mutations are more diversified in comparison with other driver mutations such as *EGFR*.^9^, ^10^ *KRAS* mutation is composed of various subtypes, which may result in differential clinical outcomes. In recent years, the *KRAS* co‐occurring genomic alterations, reported separately by researchers from MD Anderson Cancer Center and Memorial Sloan Kettering Cancer Center, defined distinctive subtypes which lead to different survival outcomes.^11^, ^12^ In our study, we aim at discovering distinctive KRAS co‐mutation subtypes in Chinese population and associated unique mutation spectrum.

## 2 METHODS

### 2.1 Patient and sample preparation

Tumor specimens, with formalin‐fixed and paraffin‐embedded, were collected from advanced NSCLC patients who underwent biopsy (Bronchoscopic biopsy or CT‐guided percutaneous pneumocentesis) at Xiangya hospital between January 2015 and December 2016. Specimens were reviewed by two independent pathologists. This study was approved by the Institutional Review Board (IRB) of Xiangya Hospital. Written informed content was obtained from every patient. All patients had not received any immune checkpoint inhibitors (ICI) therapy during follow‐up.

### 2.2 Tissue DNA extraction

DNA was extracted using QIAamp DNA FFPE tissue kit (Qiagen) according to manufacturer's instructions. The DNA concentration was measured by Qubit dsDNA assay.^13^

### 2.3 NGS library preparation

DNA shearing was performed using Covaris M220, followed by end repair, phosphorylation, and adaptor ligation. Fragments of size 200‐400 bp were selected by bead (Agencourt AMPure XP Kit, Beckman Coulter). DNA template hybridized with capture probes baits, then hybrids were again selected by magnetic beads and process to PCR amplification. A bioanalyzer high‐sensitivity DNA assay was then performed to assess the quality and size of the fragments and indexed samples were sequenced on Nextseq500 sequencer (Illumina, Inc) with pair‐end reads.

### 2.4 Capture‐based targeted DNA sequencing

Genetic profiles of all tissue samples were assessed by performing capture‐based targeted deep sequencing using the 56‐gene panel (Burning Rock Biotech Ltd.). The commercially available panel, which contains 42 oncogenes, 11 tumor suppressor gene, and three metabolically related genes, was designed by Burning Rock Biotech Ltd. DNA quality and size were assessed by high‐sensitivity DNA assay using a bioanalyzer. All indexed samples were sequenced on a NextSeq 500 (Illumina, Inc) with pair‐end reads.

### 2.5 Sequence data analysis

Sequence data were mapped to the human genome (hg19) using BWA aligner 0.7.10. Local alignment optimization, variant calling, and annotation were performed using GATK 3.2, MuTect, and VarScan. Variants were filtered using the VarScan filter pipeline, when loci with depth less than 100 filtered out. At least 5 and 8 supporting reads were needed for INDELs and SNVs to be called. According to the ExAC, 1000 Genomes, dbSNP, and ESP6500SI‐V2 database, variants with population frequency over 0.1% were grouped as single nucleotide polymorphism and excluded from further analysis. Remaining variants were annotated with ANNOVAR and SnpEff v3.6. DNA translocation analysis was performed using both Tophat2 and Factera 1.4.3.

### 2.6 Follow‐up

Patient response evaluation was done based on their follow‐up clinical data and the Response Evaluation Criteria in Solid Tumors (RECIST) criteria.^14^ The endpoint is progression‐free survival (PFS) and overall survival (OS). OS was defined as the time from date of diagnosis of advanced disease (stage IV) until date of death or last follow‐up. PFS was defined as the time from the initiation of the first‐line chemotherapy until date of progression or last follow‐up.

### 2.7 Statistical analysis

Statistical analyses were conducted using SPSS version 23 (IBM Corporation) and GraphPad Prism version 8.00 for Windows (GraphPad Software). The multivariate cox regression analysis was used to evaluate prognosis‐related factors and their hazard ratio (HR) in this cohort. The selected co‐mutation genes were ranked in frequency by multiple prior analyses among 56 gene penal. The correlations of *KRAS* subtypes and patient OS or PFS were evaluated by Kaplan‐Meier survival analysis using log‐rank test. The distribution of immune‐related genes in different KRAS co‐mutation subtypes was tested by unpaired *t* tests. *P* < .05 was considered to indicate statistical significance.

## 3 RESULTS

### 3.1 Patient characteristics

The patient characteristics were shown in Table 1. A total of 1126 Chinese advanced NSCLC patients were screened and 84 (7.46%) cases were detected with *KRAS* mutation. Of these patients with *KRAS*‐mutated advanced NSCLC, 12 patients were females and the remaining 72 patients were males. The median age at diagnosis was 51 years old (ranging from 33 to 64). Nineteen patients were nonsmokers, 65 patients were current (60 patients) or former (five patients) smokers. In these smokers, three (4%) of them had no more than 20 pack‐years smoking, 46 (54%) patients had 20‐60 pack‐years smoking, and 16 (19%) patients had more than 60 pack‐years smoking. Furthermore, 80 patients were diagnosed with adenocarcinoma, meanwhile, two patients were diagnosed with large cell carcinoma, and two patients were diagnosed with sarcomatoid carcinoma. About 29 patients had tumor located in the left lung and the remaining had tumor located in the right lung. All patients were diagnosed with stage IV disease, and the performance states ranged from ECOG 0 (72 patients) to 1 (12 patients) before treatment. All of the 84 patients received pemetrexed plus platinum as the first‐line treatment. The median PFS and OS were 14.21 weeks (IQR: 10.04‐17.93) and 20.50 weeks (IQR: 16.75‐30.25), respectively (Table 2).

**Table 1:** The clinical characteristics of KRAS‐mutated Chinese NSCLC (Stage IV)

Characteristic	N = 84 (%)	COX regression model			
		*P*_PFS_	HR (95% CI)	*P*_OS_	HR (95% CI)
Age at diagnosis		.807	(0.969, 1.042)	.068	(0.997, 1.101)
Median	51				
Range (mix to max)	33‐64				
Gender		.510	(0.554, 3.287)	.892	(0.345, 2.529)
Male	72 (86%)				
Female	12 (14%)				
ECOG PS		.617	(0.367, 1.813)	.066	(0.123, 1.068)
0	72 (86%)				
1	12 (14%)				
Smoking history		.299	(0.982, 1.006)	.128	(0.996, 1.034)
Never	19 (23%)				
Current	60 (71%)				
Former	5 (6%)				
Pack‐years					
Non	19 (23%)				
<20	3 (4%)				
20‐60	46 (54%)				
>60	16 (19%)				
Pathological types		.734	(0.264, 2.555)	.895	(0.293, 4.075)
Adenocarcinoma	80 (96%)				
Large cell carcinoma	2 (2%)				
Sarcomatoid carcinoma	2 (2%)				
Tumor position		.821	(0.460, 1.467)	.322	(0.334, 1.434)
Right	55 (65%)				
Left	29 (35%)				
Kras mutation sites		.002	(1.785, 13.637)	.839	(0.000, 1.206E+53)
p.G12X	68 (81%)				
p.G13X	12 (14%)				
p.Q61H	4 (5%)				
Kras co‐mutation		.001	(1.732, 8.647)	.012	(1.349, 11.254)
Kras mutation	24 (28%)				
Kras co‐mutation	60 (72%)				

Note

ECOG, Eastern Cooperative Oncology Group; The Kras^G12X^ mutation contains Kras^G12C^, Kras^G12D^, Kras^G12V^, Kras^G12A^, and Kras^G12R^; The Kras^G13X^ mutation contains Kras^G13C^ and Kras^G13D^.

*The *P* value was calculated using Cox regression models.

**Table 2:** The survival of different KRAS subtypes (weeks)

Subtypes	PFS (median, IQR)	Overall survival (median, IQR)
All patients	14.21 (10.04‐17.93)	20.50 (16.75‐30.25)
KRAS co‐mutation		
KP	12.86 (5.00‐15.57)	16.29 (10.04‐22.00)
KL	12.43 (10.96‐15.50)	19.36 (16.86‐24.29)
KPL	12.29 (8.50‐14.28)	20.22 (8.46‐31.43)
KK	32.71 (31.50‐33.82)	34.35 (33.75‐35.07)
KC	16.29 (15.14‐18.93)	20.57 (19.00‐22.04)
KRAS	22.29 (10.04‐30.29)	28.57 (17.50‐30.75)
KRAS mutation		
G12C	15.57 (12.39‐17.29)	18.64 (12.39‐30.75)
G12D	11.00 (8.07‐15.07)	21.35 (19.14‐25.29)
G12V	23.28 (13.79‐29.43)	27.57 (23.18‐30.39)
KRAS amino acid substitution		
G>A	11.00 (8.07‐15.07)	21.36 (19.14‐25.29)
G>C	9.71 (6.46‐12.50)	15.08 (12.68‐17.36)
G>T	15.93 (13.21‐22.04)	24.21 (16.96‐30.43)

### 3.2 The prevalence and genotype distribution of KRAS mutation and co‐mutation

**Figure 1:**
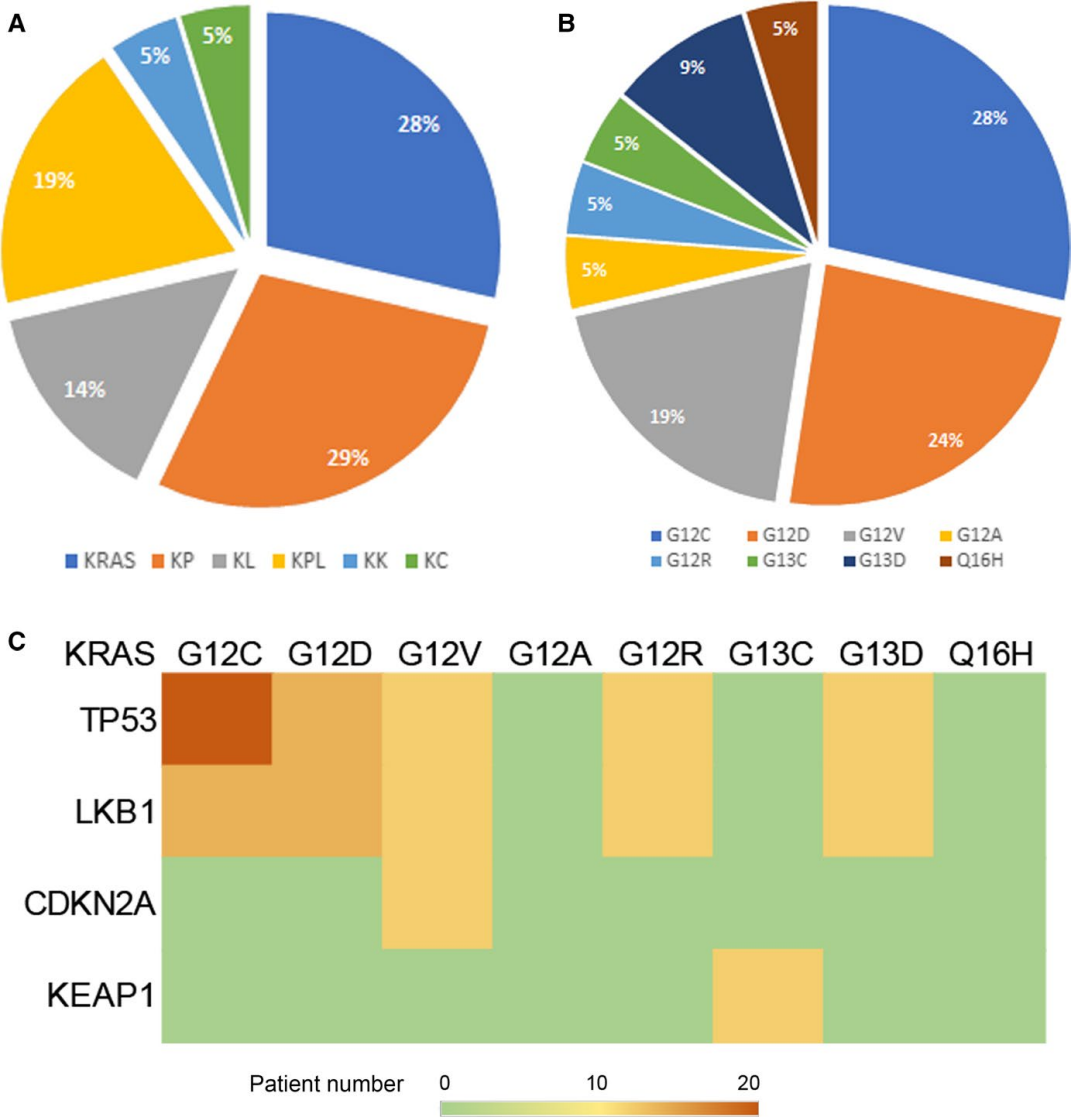
The prevalence and genotype distribution of *KRAS* co‐mutation in Chinese advanced non‐small cell lung cancer patients; A, *KRAS* co‐mutation subtypes in the cohort of 56‐genes panel; B, *KRAS* mutation sites in the cohort of 56‐genes panel; C, the concurrent mutations which occur with different KRAS mutations

The most frequently seen *KRAS* mutations included *KRAS^G12C^* (28%), *KRAS^G12D^* (24%), and *KRAS^G12V^* (19%), which account for 71% of all *KRAS* mutation cases (Figure 1B). The concomitant mutated genes belonged to non‐oncogene subpanel in 56 gene panel were ranked in frequency by multiple prior analyses (Supplement‐56 gene panel). In an agreement with previous studies, the most commonly co‐occurring genes were *TP53* (29%), *TP53/LKB1* (19%), and *LKB1* (14%). Other frequently seen co‐mutations included *KEAP1* (5%) and *CDKN2A* (5%). Subsequently, the multivariate cox regression analysis was used to identify potential risk factors in this cohort. The results revealed that KRAS co‐mutation subtypes were significantly correlated with OS and PFS. (Tables 1 and 3). The *KRAS* subtypes were further stratified into four groups according to the presence of specific co‐mutations: single *KRAS* mutation, KP (*KRAS* and *TP53* mutations), KPL (*KRAS*, *TP53*, and *LKB1* mutations), and KL (*KRAS* and *LKB1* mutations). The prevalence of each *KRAS* co‐mutation subtype is shown in Figure 1A. Little over a quarter of the patients harbored single KRAS mutation and 29% of patients harbored KRAS in combination with TP53 mutation. The concurrent mutations occurred with different KRAS mutations which appeared mostly in KRAS^G12C^ and KRAS^G12D^ sites (Figure 1C).

**Table 3:** The multivariate analyses in KRAS subtypes correlated to PFS and OS

Subtypes	_COX regression model_			
	*P*_PFS_*	HR (95% CI)	*P*_OS_*	HR (95% CI)
Kras co‐mutation				
KPL	.0001	(2.624, 26.553)	.0001	(5.590, 352.187)
KP	.571	(0.420, 4.819)	.01	(1.930, 135.404)
KL	.054	(0.980, 9.691)	.027	(1.307, 83.276)
Kras	.105	(0.155, 1.192)	.272	(0.095, 1.944)
Kras mutation sites				
G12C	.484	(0.297, 1.777)	.055	(0.026, 1.038)
G12D	.0001	(3.836, 28.145)	.01	(1.596, 30.972)
G12V	.97	(0.394, 2.629)	.059	(0.019, 1.076)

Note

P*: *P* value was calculated by Cox proportional hazards regression model, *P* < .05 was considered to indicate statistical significance.

### 3.3 Prognostic value of co‐mutation subtypes

**Figure 2:**
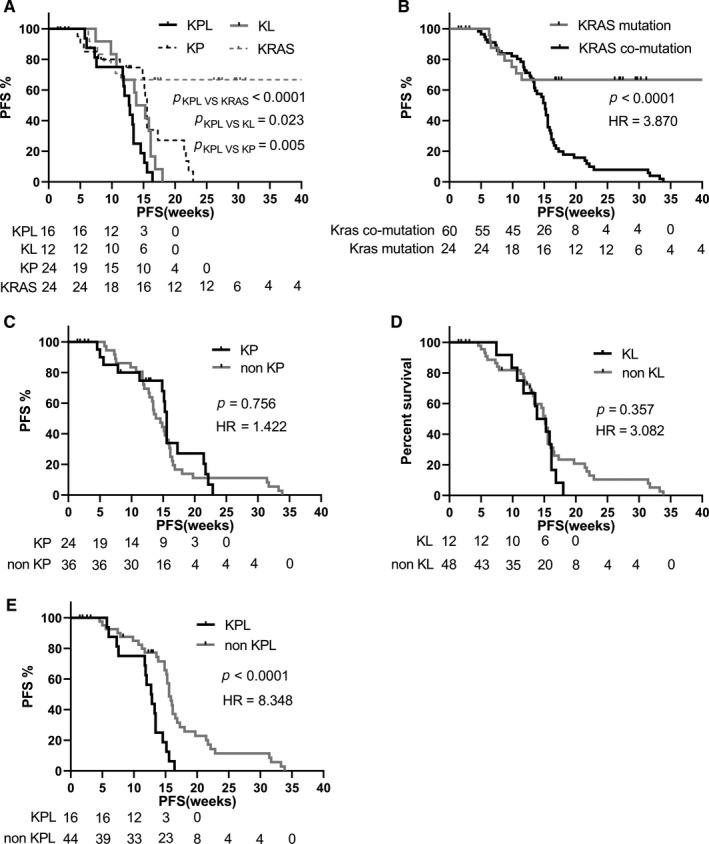
The progression‐free survival of different *KRAS* co‐mutation subtypes in Chinese advanced non‐small cell lung cancer patients. A, the PFS among KP, KL, KPL, and *KRAS* types were analyzed using Kaplan‐Meier and log‐rank test; B, the type of *KRAS* co‐mutation has shorter PFS in contrast with the ones of single *KRAS* mutation which has non‐co‐mutation with other vital genes like *TP53*, *CDKN2A*, *LKB1*, *KEAP1*; C, patients with KP mutation have similar PFS as the patient with non‐KP mutation (non‐KP = KL + KPL + KK + KC); D, the PFS of patients with KL mutation are similar to the ones of non‐KL in statistic (non‐KL = KP + KPL + KK + KC); E, the PFS of patients with KPL mutation are worse than the ones of non‐KPL mutation (non‐KPL = KP + KL + KK + KC)

**Figure 3:**
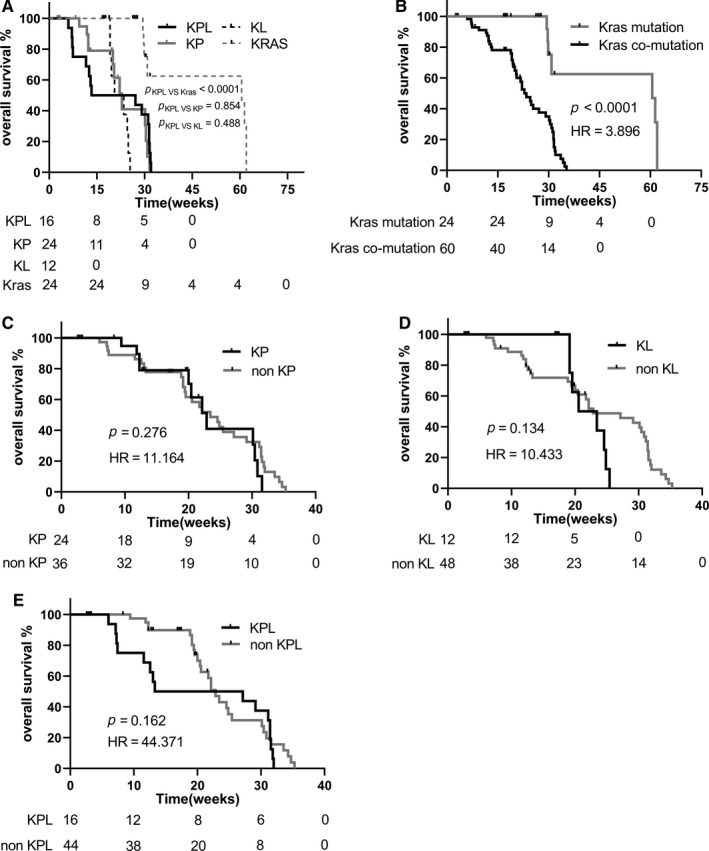
The overall survival of different *KRAS* co‐mutation subtypes in Chinese advanced non‐small cell lung cancer patients. A, the overall survival among KP, KL, KPL and *KRAS* were analyzed using Kaplan‐Meier and log‐rank test (Mantel‐Cox); B, *KRAS* co‐mutation subtypes has shorter overall survival in contrast with the ones of single *KRAS* mutation which has non co‐mutation with other vital genes like *TP53, CDKN2A, LKB1, KEAP1*; C, patients with KP mutation have similar overall survival as the patient with non‐KP mutation (non‐KP=KL+KPL+KK+KC); D, the overall survival of patients with KL mutation are similar to the ones of non‐KL in statistic (non‐KL = KP + KPL + KK + KC); E, the overall survival of patients with KPL mutation are resemblance with the ones of non‐KPL (non‐KPL = KP + KL + KK + KC)

Next, we investigated whether subtypes of KRAS co‐mutations have prognostic values. Our analysis revealed that patients with single *KRAS* mutation had statistically longer PFS and OS than those with *KRAS* co‐mutation (*P* < .0001, for both PFS and OS) (Figures 2B and 3B, Table 1). We further analyzed survival outcomes in patients with different subtypes of co‐mutations and revealed patients with KPL had the shortest PFS (Figure 2A; Table 3). Moreover, the PFS of KPL was significantly shorter than the ones of non‐KPL (Figure 2E). However, the PFS and OS of KP and KL were similar with the ones of non‐KP and non‐KL, respectively (Figures 2C,D and 3C,D).

### 3.4 Prognostic values of KRAS mutation subtypes

**Figure 4:**
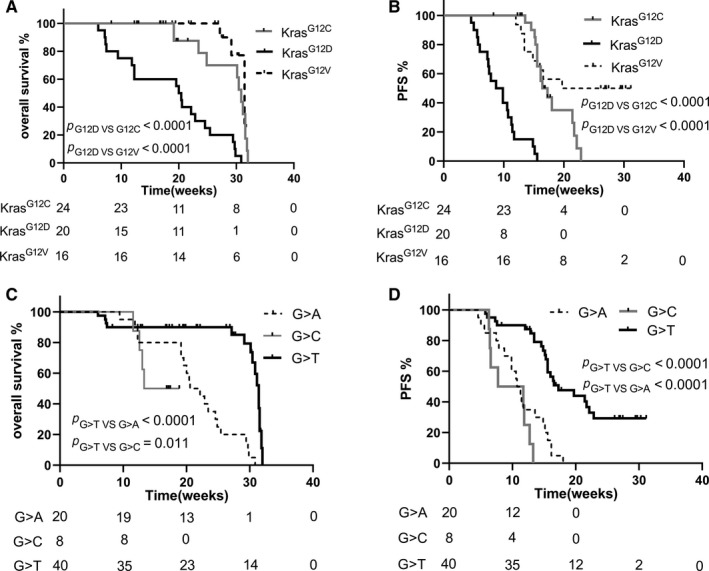
The survival of different *KRAS* mutation subtypes in Chinese advanced non‐small cell lung cancer patients. A, the comparison of overall survival in different *KRAS*‐mutated sites containing *KRAS^G12C^, KRASG^12D^, KRAS^G12V^*, the statistical significance was analyzed by the means of Kaplan‐Meier and log‐rank test (Mantel‐Cox); B, the difference of PFS in *KRAS* mutation sites was analyzed by the means similar as A; C, at the level of amino acid substitution, the comparison of overall survival in various subtypes was presented; D, the PFS of amino acid substitution subtypes was analyzed

The cases with *KRAS^G12D^* mutation had the shortest PFS and OS in comparison with *KRAS^G12C^* (*P*_PFS_ < .0001, *P*_OS_ < .0001) and *KRAS^G12V^* (*P*_PFS_ < .0001, *P*_OS_ < .0001) (Figure 4A,B). At the level of amino acid substitution, the PFS and OS of *KRAS^G>T^* group were superior to *KRAS^G>C^* group (*P*_PFS_ < .0001, *P*_OS_ = .011) and *KRAS^G>A^* (*P*_PFS_ < .0001, *P*_OS_ < .0001) (Figure 4C,D).

### 3.5 TCGA data analysis

**Figure 5:**
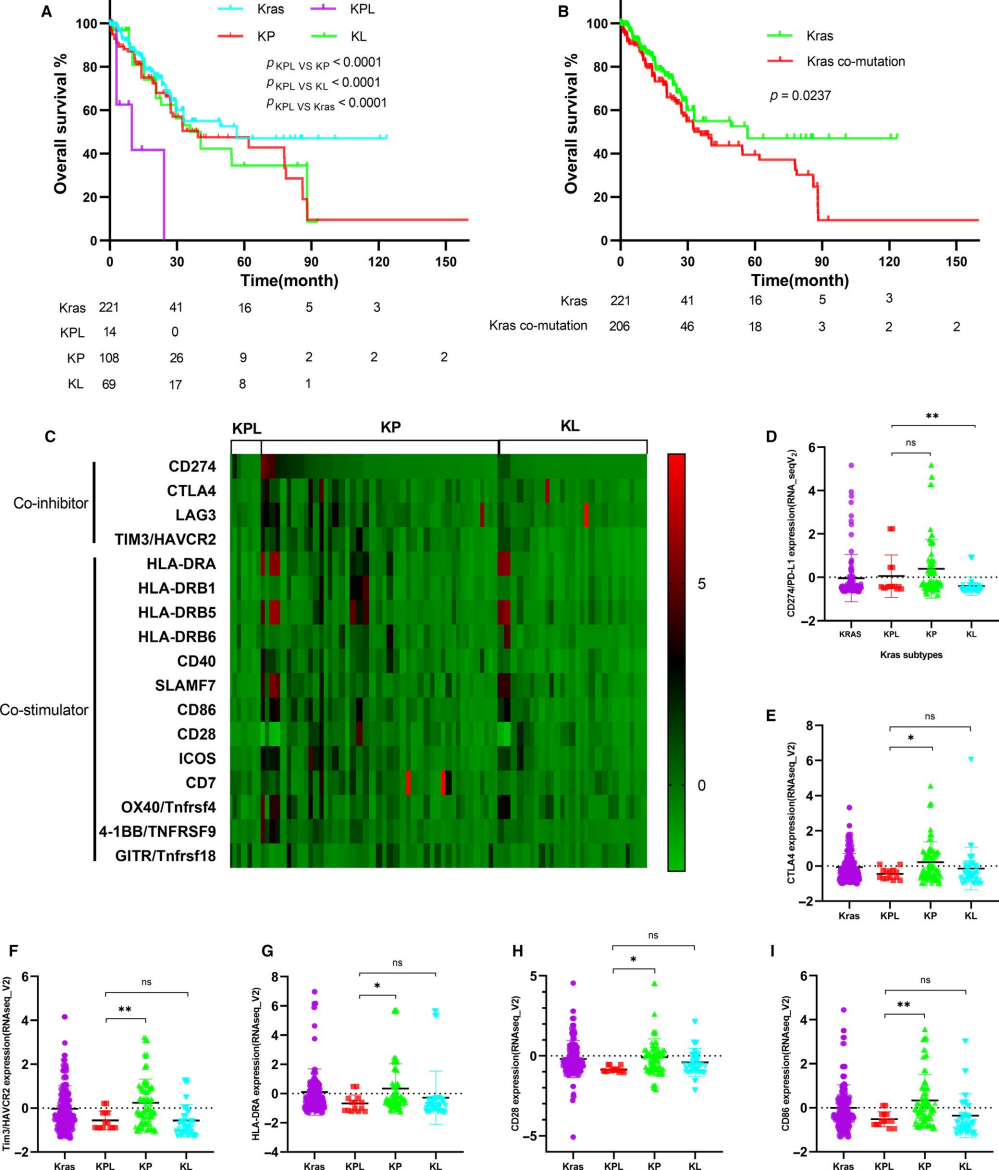
The overall survival and distinctive expression of immune‐maker genes among different *KRAS* co‐mutation types in TCGA cohort. A and B, the KPL type have poor OS Page 21 of 27 Cancer Medicine compared to other *KRAS* co‐mutation subtypes, the statistical significance was analyzed by the means of Kaplan‐Meier and log‐rank test (Mantel‐Cox). C, the heat map was built based on the expression of immune‐related genes in different *KRAS* subtypes. D‐I, with further details, except for CD274/PD‐L1 expression, the lower immune‐costimulatory and immune‐coinhibitory genes were expressing in KPL type compared to KP type. The statistical significance was analyzed by unpaired *t* tests

Next, to validate our findings, we retrieved 450 KRAS‐mutant patients with available survival data and KRAS subtypes details from the TCGA dataset. In an agreement with our findings, the OS of KPL subtype was inferior to the ones with KP, KL, or Kras types (Figure 5A,B). With matched RNAseq data in TCGA, the expression of some important molecules about tumor immunity was analyzed. Interestingly, we found that the expression of immune‐related genes was different in these Kras subtypes (Figure 5C). With further details, except for CD274/PD‐L1 expression, the lower immune‐costimulatory and immune‐coinhibitory genes were expressing in KPL type compared to KP type (Figure 5D‐I).

## 4 DISCUSSION

About 20%‐30% of NSCLC patients in Caucasian population and 8% of NSCLC patients in Asian population were observed to harbor *KRAS* mutation.^15^, ^16^, ^17^ In our cohort, 7.46% of Chinese NSCLC patients harbor *KRAS* mutation. Most of the studies investing the genomic landscape of KRAS‐mutant patients primarily consisted of non‐Chinese patients. In this study, we presented genomic landscape of distinctive *KRAS* co‐mutation subtypes and their correlation with treatment and survival outcomes.

The concurrent mutations, such as *TP53*, *STK11* (*LKB1*), *KEAP1*, and *ATM*, might contribute to the diverse response observed in *KRAS*‐mutant NSCLC.^18^ In 2015, Skoulidis et al summarized characteristics of three *KRAS* co‐mutation subtypes: KP vs KL vs KC.^12^ In 2017, Arbour et al reported the unfavorable survival of *KRAS*‐mutant patients with concurrent *KEAP1* alteration, which belonged to a new stratification: KP vs KL vs KK.^11^ In our study, we discovered a new subtype: KPL (*KRAS* mutation with *TP53* and *LKB1* mutated) which had the most unfavorable PFS among all *KRAS* mutation subtypes.

To date, the most optimal treatment of *KRAS‐*mutant lung cancer remains controversial. Before 2018, in China, pemetrexed plus platinum is still the first‐line treatment for advanced NSCLC patients. In recent years, tremendous efforts have been invested in elucidating the most optimal treatment strategy for KRAS‐mutant NSCLC patients. For instance, the KP subtype with high levels of immune score may be particularly responsive to therapeutic targets such as PD‐L1, PD‐1, and CTLA‐4. However, the KL, KK, and KC subtypes are less responsive to ICI.^11^, ^12^, ^16^ According to RNAseq data from TCGA, except for CD274/PD‐L1 expression, the lower immune‐costimulatory and immune‐coinhibitory genes were expressing in KPL type compared to KP type. More studies are needed to investigate whether immunotherapy can serve as a better choice for patients of KPL subtype. Further investigation of new anticancer regimens is still warranted for this subtype.

In our cohort, the survival of patients with *KRAS^G12D^* was shorter in comparison with *KRAS^G12C^* and *KRAS^G12V^* types. This can be potentially explained by that the GTP‐bound G12D mutation exhibits almost identical interactions as the wild‐type, while the intercation of GTP‐bound G12C or GTP‐bound G12V differred from the one of GTP‐bound G12D.^19^ Meanwhile, mutant *KRAS* proteins also affect patient survival through different downstream signaling pathways.^20^ In recent years, *Kras^G12C^* was considered as a potential druggable target; inhibitors such as ARS‐1620, MEK inhibitors, and quinazoline series have been developed.^21^, ^22^, ^23^ Especially, a case reported that a patient with synchronous *EGFR^G719S^* and *KRAS^G12C^* mutations survived for more than 9 years under treatment of erlotinib,^24^ highlighting the potential of KRAS inhibitors. At amino acid substitution levels, our results were in an agreement with Alona's study which showed that NSCLC patients of *KRAS^G>T^* substitution mutations had longer OS than that of *KRAS^G>C^*.^25^ In future clinical practice, advanced *KRAS*‐mutant patients may benefit from further stratification into different KARS subtypes.

## CONFLICT OF INTEREST

None declared.

## ETHICS APPROVAL

This study was approved by the Institutional Review Board (IRB) of Xiangya Hospital. Written informed content was obtained from every patient. The study was conducted in accordance with the Declaration of Helsinki.
